# Low-Cost CO_2_ NDIR Sensors: Performance Evaluation and Calibration Using Machine Learning Techniques

**DOI:** 10.3390/s24175675

**Published:** 2024-08-31

**Authors:** Ravish Dubey, Arina Telles, James Nikkel, Chang Cao, Jonathan Gewirtzman, Peter A. Raymond, Xuhui Lee

**Affiliations:** 1 School of the Environment, Yale University, New Haven, CT 06511, USA; jonathan.gewirtzman@yale.edu (J.G.); peter.raymond@yale.edu (P.A.R.); xuhui.lee@yale.edu (X.L.); 2 Department of Physics, Yale University, New Haven, CT 06511, USA; arina.telles@yale.edu (A.T.); james.nikkel@yale.edu (J.N.); 3 School of Applied Meteorology, Nanjing University of Information Science & Technology (NUIST), Nanjing 210044, Jiangsu, China; chang.cao@nuist.edu.cn

**Keywords:** low-cost CO_2_ sensors, collocated measurements, performance evaluation, machine learning calibration

## Abstract

The study comprehensively evaluates low-cost CO_2_ sensors from different price tiers, assessing their performance against a reference-grade instrument and exploring the possibility of calibration using different machine learning techniques. Three sensors (Sunrise AB by Senseair, K30 CO_2_ by Senseair, and GMP 343 by Vaisala) were tested alongside a reference instrument (Los Gatos precision greenhouse gas analyzer). The results revealed differences in sensor performance, with the higher cost Vaisala sensors exhibiting superior accuracy. Despite its lower price, the Sunrise sensors still demonstrated reasonable accuracy. Meanwhile, the K30 sensor measurements displayed higher variability and noise. Machine learning models, including linear regression, gradient boosting regression, and random forest regression, were employed for sensor calibration. In general, linear regression models performed best for extrapolating data, whereas decision tree-based models were generally more useful in handling non-linear datasets. Notably, a stack ensemble model combining these techniques outperformed the individual models and significantly improved sensor accuracy by approximately 65%. Overall, this study contributes to filling the gap in intercomparing CO_2_ sensors across different price categories and underscores the potential of machine learning for enhancing sensor accuracy, particularly in low-cost sensor applications.

## 1. Introduction

Carbon dioxide (CO_2_) is one of the major greenhouse gases (GHG) responsible for contemporary climate change. Studies have shown that tropospheric CO_2_ levels often exhibit significant spatial variation, which depends on land usage [1,2] and topography [3]. CO_2_ measurements can be used as a proxy to estimate carbon emissions. Monitoring these fine-scale spatial variations of CO_2_ can help to identify emission hotspots, assess local carbon cycling, track urbanization effects, and develop strategies to reduce overall carbon emissions [1,3,4].

Historically, wide spatial coverage and resolution of CO_2_ measurements has been limited by the high cost of instrumentation. Low-cost CO_2_ sensors have become increasingly popular over recent years owing to technological advancements in electronics and the Internet of Things (IoT). These sensors are generally small, portable, and low power. Usually, these sensors either use a solid electrolyte or a non-dispersive infrared (NDIR) detector [5]. NDIR sensors are more stable, accurate, and consume less power compared to sensors using solid electrolytes [6,7]. Furthermore, ease of operation and IoT capabilities make them ideal for setting up a dense network of multiple sensors to measure the distribution of CO_2_ concentrations at high spatial resolution. A dense network of reasonably accurate low-cost CO_2_ sensors can provide vital information leading to a better understanding of carbon fluxes and their sources [8]. However, obtaining accurate data from these low-cost sensors is a major challenge. Many sensors suffer from inadequate quality control and stability, leading to inconsistent performance across various conditions [9]. Over time, sensor measurements tend to drift, compromising accuracy [10]. Moreover, conducting in situ recalibration, especially within extensive sensor networks, is often impractical. Additionally, environmental variables like temperature and humidity induce non-linear responses, further complicating sensor usability [11]. Despite their cost-effectiveness, it is imperative to address these shortcomings to ensure dependable environmental monitoring.

The United States Environmental Protection Agency (EPA) over the last few years has focused on development and performance targets for particulate matter, ozone, nitrogen dioxide, sulfur dioxide, and carbon monoxide sensors [12]. It published detailed guidelines on testing protocols and target values for particulate matter and ozone sensors and is currently participating in the American Society for Testing and Materials (ASTM) study (ASTM WK74360) to develop methods for testing and evaluating CO_2_ sensors [12]. Studies that evaluate the performance of low-cost sensors help in setting the performance targets for the sensors. Past studies have evaluated low-cost CO_2_ sensors in controlled [8] and uncontrolled environments [5,7,8]. These studies have also evaluated the dependence of sensors on several factors, such as temperature, relative humidity (RH), and pressure. Further, low-cost sensors vary significantly in cost, ranging from ~35 USD to ~3500 USD. Past studies have evaluated low-cost CO_2_ sensors ranging from ~50–100 USD [13,14,15,16,17] to ~3500 USD [18]. However, the intercomparison of differences in the performance and accuracy of low-cost sensors from different price categories has not been well studied in the past, which is necessary to better understand the applicability of low-cost CO_2_ sensors. This will also contribute to US EPA and ASTM efforts in setting performance targets for CO_2_ sensors. Further, to make low-cost sensors an effective tool for research, it is required to calibrate the sensors and ensure the data reported are accurate. Several past studies have attempted to use statistical [8] and machine learning techniques [7,8,11,19] to improve the performance of sensors. However, these studies do not compare the performance differences of sensors from different price categories [20]. Our study aims to bridge this gap. This comparison will help in selecting a sensor that balances accuracy and budget, depending on the specific application. 

We evaluated three different low-cost sensors, testing three replicates of each. The low-cost sensors investigated in this study are as follows: (a) Sunrise AB by Senseair (hereafter referred to as Sunrise), (b) K30 CO_2_ by Senseair (K30), and (c) GMP 343 by Vaisala (Vaisala). The sensors’ performance evaluation is based on collocated measurements next to a reference-grade instrument, a Los Gatos Research analyzer by ABB (model GLA 131; hereafter referred to as LGR). The results from the study highlight the differences in the performance of low-cost sensors from two different price categories: (a) Tier I, Vaisala (~3500 USD) and (b) Tier II, Sunrise and K30 (~60 to 100 USD). One objective of this study is to calibrate and improve the accuracy of low-cost sensors using machine learning. Additionally, the study aims to evaluate the effectiveness of calibrating these sensors in environmentally controlled chambers. To test this method, we operated three Sunrise sensors alongside LGR in control chambers under varying conditions. The data collected were used to calibrate the sensors with machine learning techniques and test the models’ performance using ambient air measurements. While previous studies have evaluated sensor performance in controlled chambers [11,21] and indoor environments [22], to the best of our knowledge, there are no studies that assess the performance of calibration models developed in chamber settings when applied to ambient environmental data. This evaluation is crucial for understanding the applicability of chamber-based calibration of low-cost sensors. 

## 2. Methodology

### 2.1. Measurement Site

All of the measurements were made on the campus of Yale University in New Haven, CT, USA. For the control chamber tests, the collocated measurements were conducted in a temperature-, humidity-, and CO_2_-controlled chamber (model: EGC C6 Environmental Control System). The outdoor ambient air tests were performed on the rooftop of the Environmental Science Center Building (41.316 N, −72.921 W) at the university campus. The site represents the ambient CO_2_ concentrations at the center of the Yale University campus.

### 2.2. Sensors

A total of nine NDIR CO_2_ sensors were tested and evaluated.

1. Sunrise AB [23] is a miniature (33.5 mm × 19.7 mm × 11.5 mm, weight: 5 g; price ~60 USD) CO_2_ sensor that operates on ultra-low power (150 μA, 3.05–5.5 V). It has a measurement range of 400–5000 ppm and stated an accuracy of ±30 ppm or ±3% of the reading. The operation ranges for temperature and RH are 0–50 °C and 0–80%, respectively. The sensor costs ~55 USD and allows UART (Universal Asynchronous Receiver/Transmitter) and I2C (Inter-Integrated Circuit) as communication interface options.
2. K30 [24] is a small (51 mm × 58 mm × 12 mm; price ~100 USD) CO_2_ sensor that has a detection range of 0 to 5000 ppm. The accuracy provided by the manufacturer is the same as that of the Sunrise sensor. The sensor supports UART and I2C. The temperature and RH operation ranges as per the manufacturer are 0–50 °C and 0–95%, respectively. The sensor requires a 4.5 to 14.0 V DC supply, and the average current consumption is rated at 40 mA.
3. Vaisala [25] is a compact (194 mm × 55 mm × 55 mm, weight: 360 g; price ~3500 USD) CO_2_ sensor designed for outdoor use. It has an overall detection range of 0–5000 ppm. The stated accuracy is (±3 ppm + 1% of the reading) for 0–1000 ppm detection range, which makes it ~10 times more accurate than the K30 and Sunrise sensors. Vaisala also offers an IP66 rating, has a temperature operating range of −40 to +60 °C, and communication is over UART. The sensor requires an 11 to 36 V DC supply, and the power consumption varies between 1 W to 3.5 W based on the state of the optics.

A Bosch BME280 Atmospheric Sensor was deployed alongside each sensor to obtain temperature, pressure, and RH values. It has a temperature range from −40 to +85 °C and an RH range from 0 to 100%. The sensor requires a 3.3 V power supply and has a current rating of 1 mA.

### 2.3. Reference Instrument

The reference instrument used for this study is LGR-ICOS (Los Gatos Research analyzer, using patented Off-Axis Integrated Cavity Output Spectroscopy technology); model GLA131, manufactured by ABB [26]. It weighs around 6.1 kg and comes in a portable case (12 cm × 34 cm × 29.5 cm). The instrument requires 10–30 VDC or 110/240 VAC supply and the maximum power consumption reported is 35 W. Its operating temperature range is 5–45 °C and it offers a data measurement range of 0.01–10 Hz. The LGR analyzer is widely regarded as a reference standard for greenhouse gas measurements offering high sensitivity and quick response time [27,28].

### 2.4. Sensors Assembly

**Figure 1:**
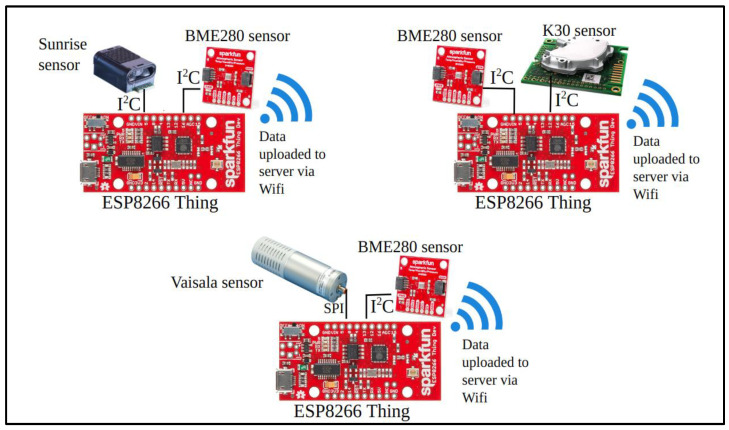
Schematic representation of the sensor assembly.

The sensors were connected to a Sparkfun ESP8266 Thing (ESP) microcontroller to acquire the data and upload it to a server via Wi-Fi. Figure 1 provides diagrams of the sensor assemblies and communications. The communication protocol between all the Sunrise, K30, and BME280 atmospheric sensors and ESP8266 is I2C, whereas the Vaisala to ESP communication protocol is SPI. The IoT network that we used for this study highlights the capability of low-cost sensors to manage and analyze the data remotely without needing physical access. This makes low-cost sensors suitable for application in dense networks to obtain high spatial resolution distribution of CO_2_. The Sunrise, K30, and BME280 sensors used I2C to communicate with ESP whereas the Vaisala used Serial UART. This approach has the advantage of managing and analyzing data remotely, which is important for dense networks aiming to obtain measurements with high spatial resolution. 

### 2.5. Experimental Setup

All of the sensors were operated directly to the LGR reference instrument. LGR was set to measure the CO_2_ concentrations at 1 s interval. The Sunrise sensors logged data every 16 s, whereas the K30 and the Vaisala sensors logged data at 30 s intervals.

**Figure 2:**
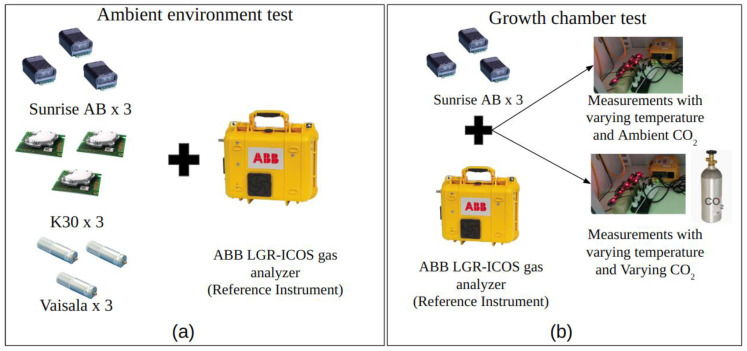
Schematic representation of the experimental setup: (**a**) ambient environment test; (**b**) growth chamber test.

For ambient environment testing, three replicates of each of the CO_2_ sensors measured the CO_2_ concentrations directly adjacent to the reference instrument LGR in the ambient environment. The measurements were made on a building rooftop at Yale University campus, as represented in Figure 2a. The measurements were made from August 8 to October 14, 2023. The CO_2_ levels varied within a range of 387 ppm to 593 ppm, with a mean concentration of 431 ppm. The mean temperature during the measurement duration was 22 °C (range: 9 °C to 38 °C) and the mean RH was 72% (range: 25% to 100%). The sky conditions were partly sunny for most of the days and were overcast for a few days.

During the controlled environment testing, three Sunrise sensors along with the reference instrument were operated adjacent to each other inside a closed environmental growth chamber (Figure 2b). The growth chamber was programmed on a 24 h cycle to set the variations in temperature from 14 °C to 32 °C. The RH in the chambers was monitored but not controlled. The growth chamber measurements were performed in two different settings of CO_2_: (i) ambient CO_2_ variations (range from 418 ppm to 850 ppm; mean 507 ppm), and (ii) controlled CO_2_ variations by injecting CO_2_ from a CO_2_ gas cylinder tank (range from 458 ppm to 871 ppm; mean 631 ppm). Figures S1 and S2 show the photos of the experimental setups at the ESC building rooftop and environmental control chambers, respectively.

### 2.6. Data Processing and Performance Evaluation

All measurements were converted into minute-wise averages to intercompare the sensor performance and calibrate using machine learning algorithms. LGR accurately measures the concentration of water vapor in the air sampled and utilizes this data to adjust the absolute (wet) CO_2_ measurements to reflect the molar mixing ratio of CO_2_ to dry air. The low-cost sensors measure the wet CO_2_ concentration (CO_2_wet_). The wet CO_2_ concentration was converted to dry CO_2_ molar mixing ratios using RH and temperature obtained from the BME280 atmospheric sensor [29]. The dry CO_2_ molar ratio (CO_2_dry_) was obtained using Equation (1), as follows:

(1) $$\frac{CO_{2_{wet}}}{V_{dry}}\cdot \frac{1013}{P}$$

where *P* is atmospheric pressure in hPa, and *V_dry_* represents the volume of 1 m^3^ dry air at 1013 hPa with no water content and it was calculated using Equation (2), as follows:

(2) $$V_{dry}=\frac{P-\left(P_{ws}\cdot \;\frac{RH}{100}\right)}{P}$$

where *P_ws_* represents saturation water vapor pressure in hPa. These corrections were applied to all of the sensor measurements before the evaluation of their performance and calibration. The corrected sensor measurements are hereafter referred to as CO_2_dry_.

The performance of the sensors was based on the parameters recommended by the US EPA for the evaluation of low-cost particulate matter and ozone sensors [30]. It recommends using slope, intercept, R^2^, bias, and RMSE (Root Mean Squared Error) values of a linear regression plot with a reference instrument as an independent variable and the low-cost sensor as a dependent variable.

### 2.7. Sensor Calibration Using Machine Learning

For the ambient environment tests, 85% of the collected data were used to train the machine learning algorithms, and the 15% of the data were used to test the model. The test data, separate from the training dataset, was used to evaluate the model’s performance. RMSE values obtained from the scatter plot between the predicted and actual values were considered the main parameter to rank the model’s performance.

Similarly, the machine learning algorithms were trained using the data from the two different types of chamber studies for the Sunrise sensors. In this case, the ambient environment measurement data from the Sunrise sensors were the test dataset.

For both tests, CO_2_dry_, RH, temperature, and pressure were used as predicting variables (input features), and the reference concentration CO_2_reference_ measured by LGR as the target variable. Equation (3) represents the regression model used for this study, as follows:

*Y* = *f*(*X*) + *ε* (3)

where *Y* is the target value i.e., CO_2_reference_; and *f(X)* represents a function that relates input features *X* to the predicted output of *Y*. Here, *X* is a vector of input features (CO_2_dry_, RH, temperature, and pressure), and *ε* is the error term representing the difference between predicted output and true output.

A total of 10 different machine learning algorithms were tested, of which the 5 best performing models were selected for analysis in this study. A brief description of the 5 selected machine learning models is as follows:

1. Multiple linear regression: This is a classic statistical technique used to model the relationship between a dependent variable and multiple independent variables. Having very low computation requirements, this simple model assumes that there exists a linear relationship between the variables [31]. The model estimates coefficients to minimize the difference between predicted and actual values.
2. Decision tree regression: This is a machine learning technique that involves splitting the whole dataset based on variables and features such that it forms tree-like structures. The predictions are made continuously by assigning a mean or weighted mean value to the leaf nodes of each tree. The model allows for intuitive interpretation. However, it may lead to overfitting if not clipped properly [32].
3. Gradient boosting regression: This is an ensemble machine learning method that builds a predictive model by combining the predictions of multiple weak learners (typically decision trees) [33]. The model reduces errors by sequentially fitting new trees to the residuals of previous trees. The final prediction is a weighted sum of individual tree predictions and not a mere average of different decision trees, resulting in a robust and accurate regression model.
4. Random forest regression: This is another ensemble machine learning technique that aggregates tree predictors that are random and independent from each other [34]. The final prediction is an average or weighted combination of individual tree predictions.
5. Stacked ensemble model: A stacked ensemble is a machine learning technique that combines predictions from multiple base models by training a meta-model on their outputs [35]. As the name suggests, the base models can be stacked one over the other and the overall model can have different levels. For this study, we used linear regression, gradient boosting regression, and random forest regression as the base or level 0 models, followed by a linear regression model as the final model, i.e., level 1 model.

## 3. Results and Discussion

### 3.1. Sensor Intercomparison

**Figure 3:**
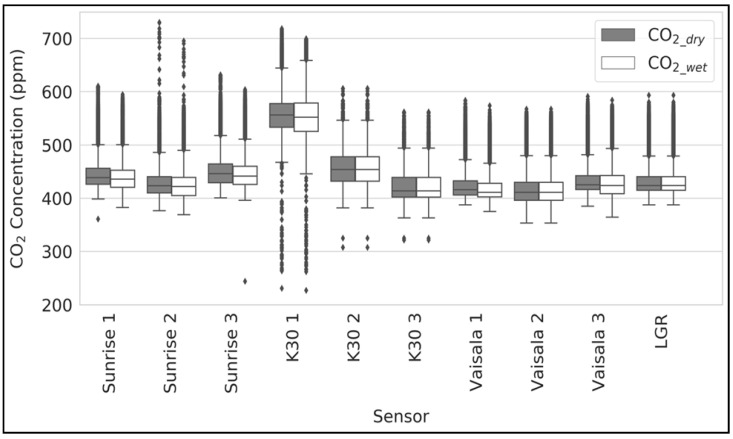
Boxplot showing the CO_2_wet_ and CO_2_dry_ values during the ambient environment measurements at the rooftop.

Figure 3 shows a boxplot comparing the ambient measurement data from all sensors and the reference instrument, both before and after applying the wet correction. The suffix numbers 1, 2, and 3 after the sensor names denote the three replicates. Table S1 shows the mean values of CO_2_wet_ and CO_2_dry_. The mean values of the CO_2_wet_ concentrations for the three replicates of the Sunrise, K30, and Vaisala sensors were 437.8 ppm, 478.2 ppm, and 421.9 ppm, respectively. The mean values of the CO_2_dry_ concentrations after applying the correction for all three Sunrise, K30, and Vaisala sensors were 442.0 ppm, 480.8 ppm and 425.6 ppm, respectively. The mean value of the dry CO_2_ concentrations from the reference instrument, i.e., LGR, was 431.8 ppm.

**Figure 4:**
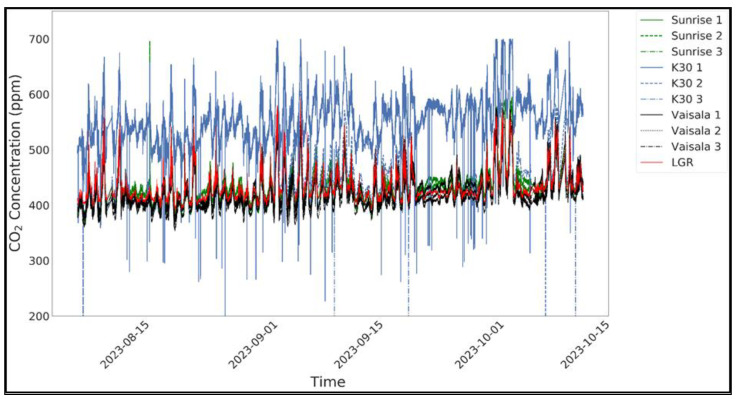
Time-series plot for the CO_2_dry_ measurements from all of the sensors and LGR during the ambient environment measurements from August 8, 2023 to October 14, 2023.

Figure 4 shows a time-series plot for all of the sensors with the reference instrument. Figure 3 and Figure 4 show that the K30 1 and K30 2 sensors overpredicted the CO_2_ concentrations, whereas K30 3 and all of the Sunrise and Vaisala sensors measured the CO_2_ values close to the LGR measurements. The K30 sensor measurements exhibited more noise, with a mean variance of 1283 ppm^2^ when compared to 898 ppm^2^ by Sunrise and 839 ppm^2^ by Vaisala.

The ambient results from the evaluation based on the linear regression plot of all of the sensors’ CO_2_dry_ values with reference to LGR are shown in Table 1. In terms of R^2^ values, the Vaisala sensors performed the best, with a combined (three replicates) mean R^2^ value of 0.92, followed by the Sunrise and K30 sensors, with mean values of 0.86 and 0.75, respectively. Based on the cost and manufacturer-claimed accuracy, these results were expected. However, considering that Vaisala costs ~60 times more than the Sunrise sensors, the performance of the Sunrise sensors was reasonable. The combined means of the slope values for the Vaisala, Sunrise, and K30 sensors were 0.92, 0.80, and 0.70, respectively.

If we observe the RMSE values, the Vaisala sensors had the lowest values (mean: 13 ppm), followed by the Sunrise (mean: 21 ppm) and K30 sensors (mean: 59 ppm). The values for the Vaisala and Sunrise sensors were close to the manufacturer-claimed accuracy of (±3 ppm +1%) and ±30 ppm of the reading, respectively. However, the RMSE values obtained for the K30 sensors were ~2 times the ±30 ppm accuracy reported by the manufacturer. The mean absolute bias values were 2.6%, 4%, and 10% for the Vaisala, Sunrise, and K30 sensors, respectively. The bias values were calculated using the DASC (Data Assessment Statistical Calculator) tool offered by the US EPA for collocated measurements of low-cost sensors and reference instruments.

### 3.2. Machine Learning Algorithms for Correction

**Figure 5:**
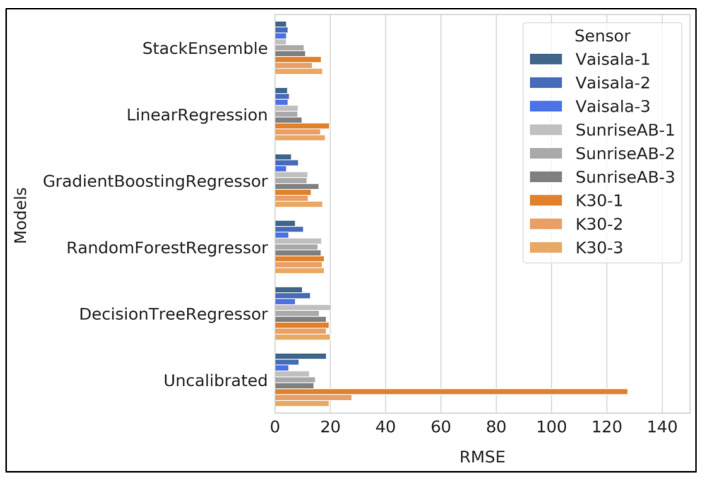
RMSE values for all three sensors and their replicates in comparison to LGR measurements before corrections and after applying corrections using different machine learning models for the testing data.

Figure 5 shows the performance of the five machine learning models for each replicate of the Vaisala, Sunrise and K30 sensors. The RMSE values improved significantly after applying the corrections.

The stack ensemble model was the best-performing model overall (mean RMSE 9.53 ppm), followed by linear regression (mean RMSE 10.53 ppm), gradient boosting regression (mean RMSE 11.09 ppm), random forest regression (mean RMSE 13.79 ppm), and decision tree regression (mean RMSE 15.87 ppm). The overall mean RMSE before calibration for the nine sensors was 27.57 ppm, which significantly improved by ~65% after applying the corrections using the stack ensemble machine learning model. The findings from previous studies indicated a substantial improvement in the accuracy of low-cost CO_2_ sensors, with error reductions of 90–98% [19] and an accuracy rate of 98.9% [11]. The significant error reduction observed in these studies can be attributed to the data splitting techniques employed. Our study used a data splitting method in which the testing data were entirely unseen by the model, leading to a more realistic assessment of the sensor’s performance. By contrast, prior studies often used random data-splitting methods, which can inflate performance metrics due to potential data leakage between training and testing sets [36].

For the Vaisala sensors, the mean RMSE value for the uncalibrated CO_2_dry_ measurement was 10.73 ppm, which improved to 4.3 ppm post-correction from the stack ensemble machine learning model. Similarly, for the Sunrise and K30 sensors, the mean RMSE values improved from 13.70 ppm to 8.5 ppm and from 58.27 ppm to 15.77 ppm, respectively. These results indicated that the machine learning techniques significantly improved the sensors’ overall accuracy.

**Figure 6:**
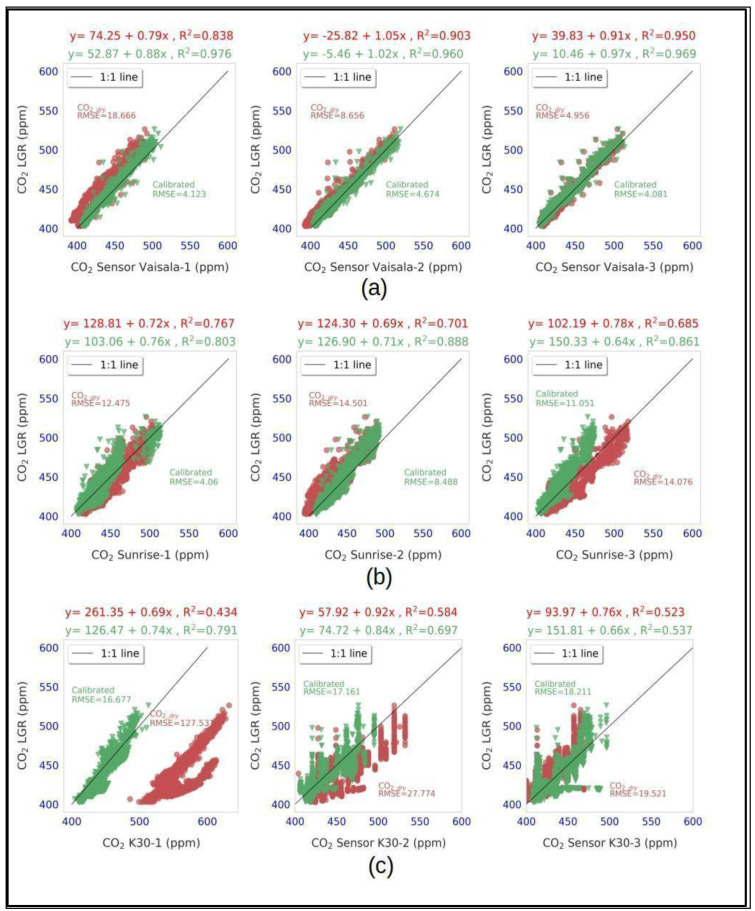
LGR vs. (**a**) Vaisala (**b**) Sunrise, and (**c**) K30 sensors showing uncalibrated CO_2_dry_ (red dots and red equation) and calibrated CO_2_ (green dots and green equation) using stack ensemble machine learning for testing data.

Figure 6 shows the scatterplot between LGR and measurements from all of the sensors before and after applying the corrections using the trained stack ensemble machine learning model for the testing dataset. In addition to the RMSE values, the machine learning model corrections resulted in improvements of the mean R^2^ values for the Vaisala, Sunrise, and K30 sensors by 8.5%, 19.2%, and 34.6%, respectively. The measurements from the K30 1 sensor notably deviated from those of the reference instrument and other sensors; its uncalibrated readings had an RMSE value of 127 ppm. However, calibration led to a substantial decrease of approximately 87% in the RMSE value. Furthermore, the uncalibrated data exhibited two distinct clusters, which were successfully eliminated through calibration (bottom left panel, Figure 6).

Linear regression models are usually better at extrapolation than decision tree-based models such as random forest regression and gradient boosting regression [37]. Random forest and gradient boosting regression models are usually better at handling non-linear datasets. Stack ensemble models enhance predictive performance by leveraging diverse model strengths and mitigating individual weaknesses, resulting in a more robust and accurate overall prediction [35]. Instead of applying individual machine learning models, we recommend using ensemble machine learning models to improve the overall calibration and achieve better performance than that achieved using individual models.

### 3.3. Environmental Chamber Measurements as Training Data

**Figure 7:**
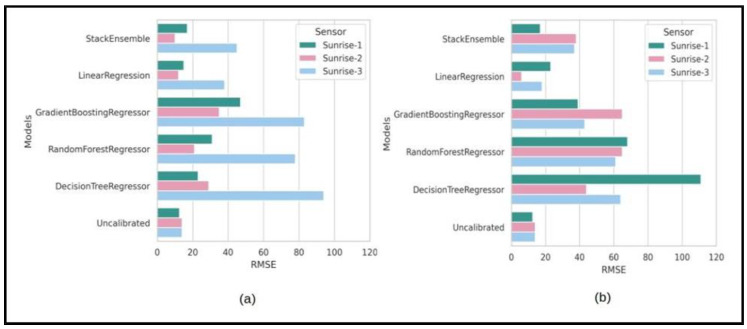
RMSE values for Sunrise sensors after calibration using different machine learning models for testing data using the training dataset: (**a**) ambient CO_2_ variations in growth chamber; (**b**) controlled CO_2_ variations in growth chamber.

The main objective of growth chamber measurements is to evaluate the possibility of applying corrections based on controlled indoor lab measurements and test the performance of the machine learning models on outdoor ambient environment datasets. In-situ/outdoor collocated measurements for calibrations are not always feasible, especially if there are many sensors and the weather conditions are not favorable. For example, if a research project requires the sensors to be deployed in summer, the growth chamber can be set up to simulate summer conditions in the winter prior to summer deployment. Figure 7 shows results for the RMSE values after applying the corrections using growth chamber collocated measurement data to train different machine learning models for the three replicates of Sunrise sensors. For both datasets, i.e., (a) ambient CO_2_ variations in the growth chamber and (b) controlled CO_2_ variations in the growth chamber, linear regression performed the best with mean RMSE values of 21.6 ppm and 15.6 ppm, respectively. However, the mean RMSE value for the uncalibrated CO_2_dry_ measurements was 13.49 for the testing dataset (ambient environments measurements at the rooftop). This means that the corrections using the machine learning models trained on the growth chamber measurements generally made the performance of the sensors worse. Only one Sunrise sensor (Sunrise #2) showed improved performance: its RMSE value decreased from 12 ppm before calibration to 6 ppm after calibration (Figure 7b). We speculate that the main reason is the differences in the overall distribution of the training dataset and testing data. Figure S3 highlights the differences in the distribution of the datasets used to train and test the machine learning models. We attempted to truncate the data from the chamber CO_2_ measurements to align with the ambient environment measurements at the rooftop, intending to utilize it for training purposes. Despite this, the performance of the machine learning models did not show improvement, suggesting that environmental conditions, such as humidity, temperature, and air pressure, also matter.

### 3.4. Limitations and Future Recommendations

Outdoor measurements were conducted from 8 August to 14 October 2023 due to the operating range of the reference instrument, LGR, which is 5 to 45 °C. Temperatures in New Haven, the study location, fall below 5 °C after October, making it impractical to continue measurements beyond this period. Additionally, the low-cost sensors used in this study, Sunrise AB and K30, have an operating range of 0 to 50 °C, further constraining the study duration. We recognize that collecting data throughout the year would likely reveal seasonal variations in the performance of low-cost sensors, and we acknowledge this limitation. We recommend future studies to test and evaluate the performance of low-cost sensors for longer duration.

Due to logistical constraints, we were unable to operate other sensors during the chamber tests, resulting in data obtained exclusively from the Sunrise sensors. This limitation could be addressed in future studies by incorporating additional sensors during testing.

Furthermore, time constraints and feasibility issues prevented us from testing the stacked ensemble model’s performance across other low-cost CO_2_ sensors. We have identified this as an area for future work and recommend exploring additional low-cost CO_2_ sensors, such as MH-Z19B, Telaire T6703, and SCD30.

## 4. Conclusions

The paper presents a detailed performance evaluation of low-cost CO_2_ sensors from different tiers of price categories (Sunrise about 50 USD; K30 about 100 USD; Vaisala about 3500 USD). The results show that the Sunrise sensors, despite their price, are fairly accurate when compared to the reference instrument LGR. The measurements from the Sunrise sensors differ from the reference instrument within the manufacturer’s claimed accuracy. Vaisala sensors offer better accuracy, but this improvement comes at a much higher cost, which may prohibit some applications. K30 sensor-reported concentrations exhibited an overall offset and the noise in their measurements was higher in comparison to the Sunrise and K30 sensors.

We further developed calibration procedures for the low-cost CO_2_ sensors using different machine learning techniques, with a stack ensemble machine learning model used to incorporate the benefits of linear regression, decision tree-based, and gradient boosting techniques. The stack ensemble model outperformed the individual models, leading to an overall reduction of 65% in the RMSE values; we recommend using this technique to calibrate low-cost CO_2_ sensors in future applications. Controlled growth chamber measurements were used to train the machine learning models and test the models’ performance for the outdoor ambient environment measurements. The models trained using the growth chamber data did not improve the sensor performance, which can be attributed to the difference in the distributions of environmental conditions within the training and testing data we used in this study. However, further study is needed to test the possibility of using controlled indoor measurements to calibrate low-cost CO_2_ sensors by imitating outdoor ambient environmental conditions.

Overall, we add to the growing body of literature on the feasibility and efficacy of low-cost CO_2_ sensors for high-density spatial resolution monitoring, particularly when cost limitations are of concern. We evaluated the performance of ultra-low-cost sensors relative to a mid-range option and a standard high-precision analyzer. The results of our study demonstrate that a range of hardware options, coupled with appropriate machine learning correction techniques, promise to enable widespread, low-cost, and high spatial resolution sensing of CO_2_ for policy-relevant monitoring applications in environmental and human health.

## Figures and Tables

**Table 1:** Evaluation of CO_2_dry_ values based on linear regression against the LGR reference instrument.

Sensors	Intercept	Slope	R Square	RMSE (ppm)	Bias (%)
Sunrise 1	62.69 ± 0.98	0.82 ± 0.0	0.86	21.49	4.01
Sunrise 2	69.26 ± 0.93	0.84 ± 0.0	0.86	12.07	±2.22
Sunrise 3	84.06 ± 1.0	0.76 ± 0.0	0.85	29.27	5.62
K30 1	77.0 ± 1.52	0.64 ± 0.0	0.71	131.47	22
K30 2	85.88 ± 1.18	0.83 ± 0.0	0.79	19.25	4.1
K30 3	143.11 ± 1.13	0.65 ± 0.0	0.74	25.3	−4.4
Vaisala 1	90.58 ± 0.81	0.83 ± 0.0	0.88	23.43	−5.14
Vaisala 2	16.31 ± 0.89	0.97 ± 0.0	0.9	9.99	±1.55
Vaisala 3	20.1 ± 0.47	0.95 ± 0.0	0.98	5.66	±1.04

## Data Availability

The data are available upon request to the authors.

## Supplementary Materials

The following supporting information can be downloaded at: https://www.mdpi.com/article/10.3390/s24175675/s1, Figure S1: Experimental setup at the ESC building rooftop; Figure S2: Experimental setup at the growth chambers with the Sunrise sensors and reference instrument; Figure S3: Violin plot showing the growth chamber training and ambient environment testing data; Table S1: Mean values of CO_2_wet_ and CO_2_dry_ concentrations.
