# Supplementary material for: Low-Cost CO2 NDIR Sensors: Performance Evaluation and Calibration Using Machine Learning Techniques
Source: Sensors (Basel). 2024 Aug 31;24(17):5675. doi: 10.3390/s24175675 (PMC11397870; doi:10.3390/s24175675)
Supplement: Supplementary file 1 [file sensors-24-05675-s001.zip › sensors-3123214-supplementary.pdf]

**Supplementary Section**

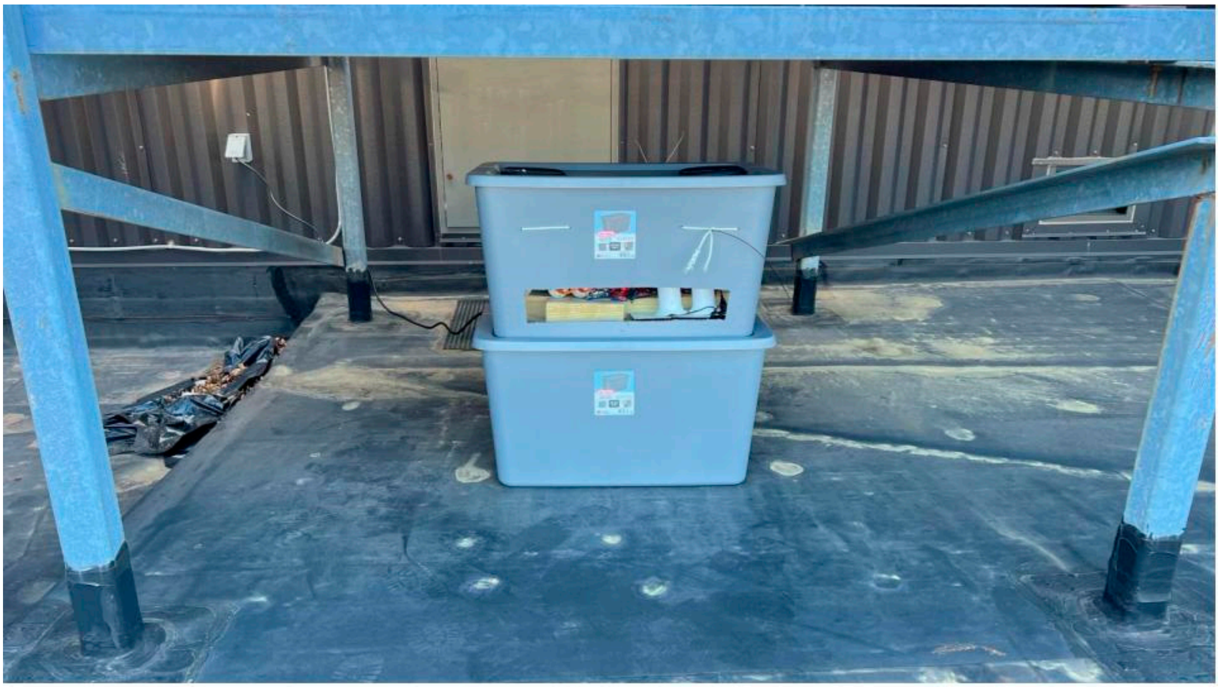

Figure S1. Experimental setup at the ESC building rooftop.

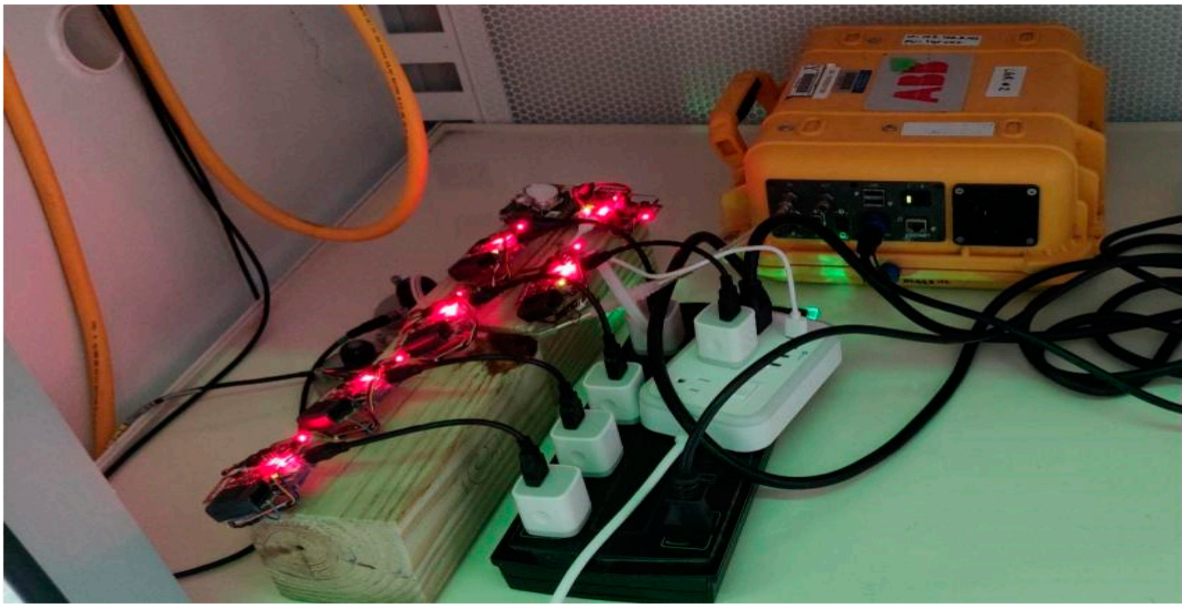

Figure S2. Experimental setup at the growth chambers with the Sunrise sensors and reference instrument.

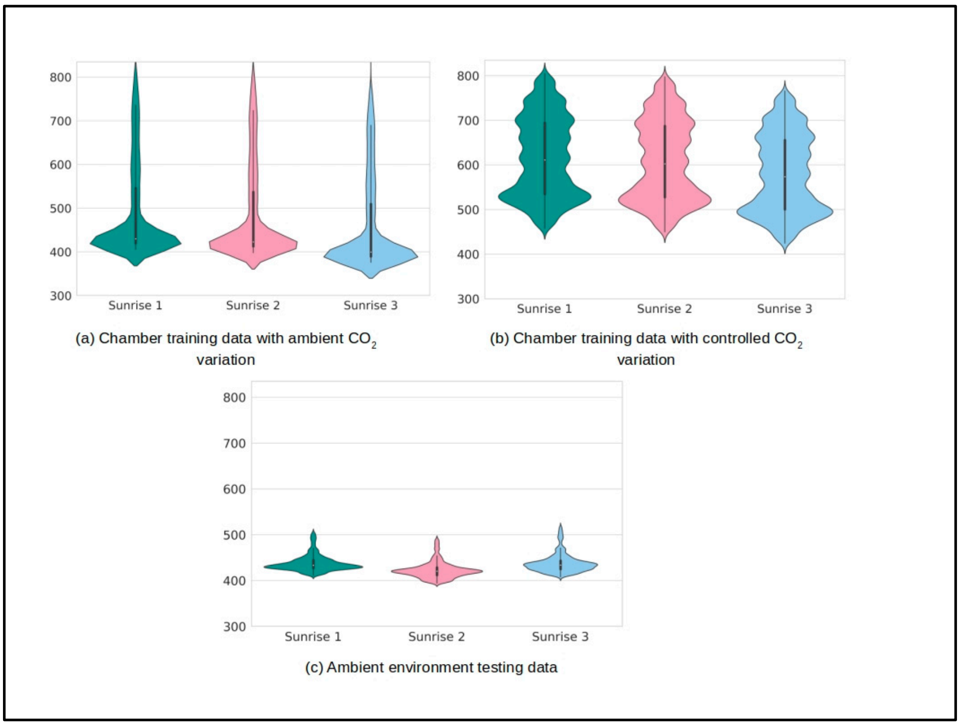


Figure S3. Violin plot showing the growth chamber training and ambient environment testing data

Table S1. Mean values of $CO_{2\_wet}$ and $CO_{2\_dry}$ concentrations

| **Sensors** | **$CO_{2\_wet}$ (ppm)** | **$CO_{2\_dry}$ (ppm)** |
|---|---|---|
| **Sunrise 1** | 440.27 | 445.59 |
| **Sunrise 2** | 426.12 | 429.39 |
| **Sunrise 3** | 446.94 | 451.01 |
| **K30 1** | 553.95 | 558.81 |
| **K30 2** | 457.84 | 459.84 |
| **K30 3** | 422.69 | 423.69 |
| **Vaisala 1** | 419.26 | 423.81 |
| **Vaisala 2** | 416.86 | 419.82 |
| **Vaisala 3** | 429.51 | 433.19 |
| **LGR** | - | 431.75 |
